# Weight change after smoking cessation and incident metabolic syndrome in middle-aged Korean men: an observational study

**DOI:** 10.1038/s41598-019-39811-0

**Published:** 2019-02-28

**Authors:** Kyuwoong Kim, Seulggie Choi, Jong-Koo Lee, Ji-Yeob Choi, Aesun Shin, Sue Kyung Park, Daehee Kang, Sang Min Park

**Affiliations:** 1 Department of Biomedical Sciences, Seoul National University Graduate School, Seoul, Republic of Korea; 2 Department of Family Medicine, College of Medicine, Seoul National University, Seoul, Republic of Korea; 3 JW Lee Center for Global Medicine, College of Medicine, Seoul National University, Seoul, Republic of Korea; 4 Department of Preventive Medicine, College of Medicine, Seoul National University, Seoul, Republic of Korea; 5 Department of Environmental Medicine, Seoul National University Medical Research Center, Seoul, Republic of Korea

## Abstract

We aimed to examine the effect of weight change attributable to cessation of cigarette smoking on newly diagnosed metabolic syndrome (MetS). We prospectively followed 5,809 men aged between 40 to 69 years without MetS at baseline in the Health Examinees-Gem (HEXA-G) study up to 4 years. The participants were grouped into continual smokers, quitters with weight gain, quitters without weight change, quitters with weight loss, and never smokers. We constructed multivariable logistic regression models adjusted for sociodemographic factors, health status, and health conditions to estimate the odds of newly diagnosed MetS. During the follow-up, there were 609 cases of newly diagnosed MetS in 5,809 men of the HEXA-G study. After adjustment for potential confounders, the odd ratios (OR) and 95% confidence intervals (95% CI) for MetS were 1.90 (95% CI: 1.43–2.52) in quitters with weight gain, 0.77 (95% CI: 0.60–1.00) in quitters without weight change, and 0.40 (95% CI: 0.28–0.57) in quitters with weight loss compared with continual smokers. Never smokers also had lower odds of MetS (OR = 0.54; 95% CI: 0.42–0.71) compared to continual smokers. Weight management program following smoking cessation may be necessary in clinical practice to reduce worsening of cardiometabolic risk factors related to post-cessation weight gain.

## Introduction

Although adverse health outcomes of cigarette smoking have been extensively studied^1–7^, weight gain after cessation of smoking still remains as one of the leading concerns among current smokers that may discourage them from quitting smoking^8–10^. Abstinence from cigarette smoking can lead to risk reduction of metabolic syndrome (MetS) through multiple pathways^11,12^, but weight gain after smoking cessation may attenuate the protective association of quitting smoking with MetS^13,14^. An up-to-date meta-analysis of prospective cohort studies has reported that smoking cessation was associated with 2.61 kg of weight gain as compared with continual smoking^4^. Since weight gain is also associated with several components of MetS^15–17^, whether weight change associated with smoking cessation counteracts the cardiometabolic benefits of quitting smoking is unclear.

Previous studies on smoking cessation and MetS have shown inconsistent results by the duration of smoking abstinence. One recent study by Song *et al*. reported that smoking cessation within 3 months is associated with increased risk of developing MetS. In contrast, others have found that smoking cessation is associated with decreased risk of MetS^18^. However, these studies were restricted to those with nicotine replacement therapy with a relatively small number of patients. Also, these studies did not take weight change attributable to smoking cessation into account. One study of 5,407 men using hospital data found that ex-smokers had elevated risk for incident MetS regardless of weight change compared to non-smokers after 3 years of follow-up^19^. Findings from these studies are subject to selection bias, misclassification of reference category, inconsistent study design, and follow-up duration.

We aimed to evaluate the association between smoking cessation, 4-year weight change, and newly diagnosed MetS and its components among the middle-aged men. We hypothesized that weight change associated with smoking cessation does not affect the risk reduction of MetS compared to continual smoking, and also investigated this association among quitters by the degree of weight change.

## Results

### Primary analysis

Among the 5,809 participants of the HEXA-G study included in our analysis, there were 1,325 (22.8%) continual smokers, 576 (9.9%) quitters with weight gain, 1,522 (26.2%), 697 (12.0%) quitters without weight change, and 1,689 (29.1%) never smokers. Of these study population, 609 participants were newly diagnosed with MetS during the follow-up period that lasted up to 4 years. Continual smokers had the highest proportion of 40–49 age group compared to quitters and never smokers. Sociodemographic, health status, and clinical characteristics of the study participants by change in smoking behavior are presented in Table 1.

**Table 1:** Sociodemographic, health status, and clinical characteristics of men by change in smoking behavior in the Health Examinees-Gem Study.

	Continual Smokers *(N* = *1,325)*	Smoking Cessation			Never Smokers *(N* = *1,689)*
		Quitters with weight gain^a^ (*N* = 576)	Quitters without weight change^b^ (*N* = *1,522*)	Quitters with weight loss^c^ (*N* = 697)	
**Age, years**					
40–49	577 (43.5)	160 (27.8)	327 (21.5)	142 (20.4)	422 (25.0)
50–59	488 (36.8)	228 (39.6)	577 (37.9)	263 (37.7)	624 (36.9)
60–69	251 (18.9)	177 (30.7)	590 (38.8)	269 (38.6)	601 (35.6)
≥70	9 (0.8)	11 (1.9)	28 (1.8)	23 (3.3)	42 (2.5)
**Education Level**					
Elementary	105 (7.9)	50 (8.7)	104 (6.8)	47 (6.7)	132 (7.8)
Middle/High School	708 (53.4)	309 (53.7)	753 (49.5)	357 (51.2)	765 (45.3)
College or Above	512 (38.7)	217 (37.6)	665 (43.7)	293 (42.1)	792 (46.9)
**Household Income**					
1^st^ Quartile	88 (6.6)	35 (6.1)	118 (7.8)	51 (7.3)	124 (7.3)
2^nd^ Quartile	263 (19.9)	119 (20.6)	311 (20.4)	132 (18.9)	297 (17.6)
3^rd^ Quartile	590 (44.5)	260 (45.1)	691 (45.4)	321 (46.1)	735 (43.5)
4^th^ Quartile	384 (29.0)	162 (28.2)	402 (26.4)	193 (27.7)	533 (31.6)
**Fagerström score**					
0	188 (14.2)	—	—	—	—
1	178 (13.4)	—	—	—	—
2	128 (9.7)	—	—	—	—
≥3	831 (62.7)	—	—	—	—
Regular Physical Activity^d^	620 (46.8)	348 (60.4)	1,018 (66.9)	445 (63.9)	1,102 (65.3)
**Alcohol Consumption**					
Non Drinker	193 (14.6)	107 (18.6)	270 (17.7)	131 (18.8)	571 (33.8)
Abstainer	46 (3.5)	58 (10.1)	137 (9.0)	75 (10.8)	109 (6.5)
Current Drinker	1,086 (81.9)	411 (71.4)	1,115 (73.3)	491 (70.4)	1,009 (59.7)
**Body Mass Index, kg/m** ^**2**^					
18.5–24.9	901 (70.1)	394 (70.1)	1,019 (69.1)	383 (56.2)	1,099 (67.7)
25.0–29.9	371 (28.8)	159 (29.3)	446 (30.2)	286 (41.9)	501 (30.9)
≥30	13 (1.1)	9 (1.6)	10 (0.7)	13 (1.9)	23 (1.4)
Waist Circumference, cm, Median (IQR)	83.0 (78.1 to 87.8)	84.0 (80.0 to 88.0)	84.0 (79.8 to 88.0)	86.0 (81.0 to 89.2)	84.0 (79.0 to 88.0)
Triglyceride, mg/dL, Median (IQR)	121.0 (86.0 to 167.0)	105.0 (76.0 to 143.5)	107.5 (77.0 to 147.0)	113.0 (81.0 to 155.0)	99.0 (71.0 to 136.0)
HDL-Cholesterol, mg/dL Median (IQR)	49.0 (43.0 to 57.0)	50.0 (43.0 to 58.0)	51.0 (44.0 to 59.0)	49.0 (43.0 to 56.0)	49.0 (44.0 to 58.0)
Systolic Blood Pressure, mmHg, Median (IQR)	120.0 (112.0 to 130.0)	122.0 (114.0 to 130.0)	123.0 (115.0 to 132.0)	124.0 (116.5 to 132.0)	123.0 (115.0 to 132.0)
Diastolic Blood Pressure, mmHg, Median (IQR)	77.0 (70.0 to 82.0)	76.8 (70.0 to 80.3)	77.5 (70.0 to 82.0)	78.0 (70.5 to 83.0)	78.0 (70.0 to 82.5)
Fasting Serum Glucose, mg/dL, Median (IQR)	92.0 (86.0 to 100.0)	93.0 (87.0 to 101.0)	94.0 (87.0 to 102.0)	95.0 (88.0 to 103.0)	93.0 (87.0 to 101.0)
Total Energy Intake, kcal/day, Median (IQR)	1,793 (1,530 to 2,133)	1,756 (1,485 to 2,075)	1,766 (1,530 to 2,057)	1,765 (1,510 to 2,050)	1,741 (1,488 to 2,062)
Family history of type 2 Diabetes	230 (17.3)	90 (15.6)	267 (17.5)	110 (15.8)	228 (13.5)
Family history of hypertension	315 (23.7)	165 (28.7)	442 (29.0)	183 (26.3)	477 (28.2)
Family history of hyperlipidemia	16 (1.2)	6 (1.0)	20 (1.3)	8 (1.2)	20 (1.2)

NOTES: Data presented as n (%) with appropriate units unless otherwise stated. Residual method was used to compute nutrient intake adjusted for energy intake.

^a^Quitters with weight gain of 2 kg (>+2 kg).

^b^Quitters with weight gain or loss of 2 kg.

^C^Quitters with weight loss of 2 kg (<−2 kg).

^d^Those who reported to routinely exercise until sweating on regular basis.

Abbreviations: IQR, Interquartile Range.

In the multivariable logistic regression analyses, weight gain following smoking cessation was associated with higher odds of elevated WC (OR = 2.94; 95% CI: 2.17–3.99) and high BP (OR = 1.72; 95% CI: 1.31–2.26). No weight change after smoking cessation was associated with lower odds of high TG (OR = 0.65; 95% CI: 0.54–0.78) and reduced HDL-C (OR = 0.53; 95% CI: 0.38–0.74). Post-cessation weight loss was associated with lower odds of elevated WC (OR = 0.34; 95% CI: 0.20–0.58), high TG (OR = 0.35; 95% CI: 0.27–0.46), and reduced HDL-C (OR = 0.56; 95% CI: 0.37–0.85). As compared with continual smokers, never smokers had lower odds of high TG (OR = 0.41; 95% CI: 0.34–0.50), reduced HDL-C (OR = 0.59; 95% CI: 0.43–0.81), and impaired FSG (OR = 0.66; 95% CI: 0.50–0.87). The association between change in smoking behavior, weight change, and newly diagnosed components of MetS are summarized in Table 2.

**Table 2:** Association of change in smoking behavior and weight change with newly diagnosed metabolic syndrome components in men in the Health Examinees-Gem Study.

	Continual Smokers	Smoking Cessation			Never Smokers
		Quitters with weight gain^a^	Quitters without weight change^b^	Quitters with weight loss^c^	
**Elevated WC** ^**a**^					
No. of participants	1,123	482	1,293	532	1,420
No. of events (%)	101	105	126	17	130
Age-adjusted Model	1 (referent)	2.73 (2.02–3.68)^***^	1.04 (0.79–1.38)	0.32 (0.19–0.54)^***^	0.97 (0.74–1.28)
Multivariable Model	1 (referent)	2.94 (2.17–3.99)^***^	1.11 (0.83–1.48)	0.34 (0.20–0.58)^***^	1.03 (0.77–1.37)
**High TG** ^**b**^					
No. of participants	901	434	1,120	496	1,347
No. of events (%)	228	129	191	69	193
Age-adjusted Model	1 (referent)	1.35 (1.04–1.74)^*^	0.68 (0.54–0.84)^**^	0.54 (0.40–0.73)^***^	0.54 (0.44–0.67)^***^
Multivariable Model	1 (referent)	1.10 (0.87–1.39)	0.65 (0.54–0.78)^***^	0.35 (0.27–0.46)^***^	0.41 (0.34–0.50)^***^
**Reduced HDL-C** ^**c**^					
No. of participants	1,126	477	1,331	596	1,484
No. of events (%)	111	72	96	41	131
Age-adjusted Model	1 (referent)	1.36 (0.97–1.93)	0.54 (0.39–0.75)^***^	0.63 (0.42–0.94)^*^	0.64 (0.48–0.87)^**^
Multivariable Model	1 (referent)	1.36 (0.96–1.94)	0.53 (0.38–0.74)^***^	0.56 (0.37–0.85)^**^	0.59 (0.43–0.81)^**^
**High BP** ^**d**^					
No. of participants	920	378	978	441	1,052
No. of events (%)	205	128	251	106	290
Age-adjusted Model	1 (referent)	1.72 (1.32–2.24)^***^	1.14 (0.91–1.41)	1.03 (0.79–1.35)	1.27 (1.03–1.56)^*^
Multivariable Model	1 (referent)	1.72 (1.31–2.26)^***^	1.13 (0.90–1.41)	0.90 (0.68–1.19)	1.31 (1.06–1.63)^*^
**Impaired FSG** ^**e**^					
No. of participants	1,173	513	1,330	590	1,449
No. of events (%)	133	69	160	70	121
Age-adjusted Model	1 (referent)	1.16 (0.84–1.59)	1.00 (0.78–1.28)	0.98 (0.72–1.34)	0.67 (0.51–0.87)^**^
Multivariable Model	1 (referent)	1.13 (0.82–1.57)	1.00 (0.77–1.29)	0.87 (0.63–1.20)	0.66 (0.50–0.87)^**^

Data presented above as adjusted OR (95% CI) using multivariable logistic regression.

^a^Quitters with weight gain of 2 kg (>+2 kg).

^b^Quitters with weight gain or loss of 2 kg.

^C^Quitters with weight loss of 2 kg (<−2 kg).

^d^Defined as waist circumference (WC) ≥ 90 cm. Not adjusted for weight change to compute OR of the elevated WC due to the high collinearity between the two variables.

^e^Defined as triglyceride (TG) ≥ 150 mg/dL.

^f^Defined as high density lipoprotein-cholesterol (HDL-C) < 40 mg/dL or treatment with antihyperlipidemic medication.

^g^Defined as systolic/diastolic blood pressure (BP) ≥ 130/85 mmHg or treatment with antihypertensive medication.

^h^Defined as fasting serum glucose (FSG) ≥ 110 mg/dL or treatment with antihyperglycemic medication.

NOTE: Criteria for the components of metabolic syndrome was defined by the National Cholesterol Education Program Adult Treatment Panel III modified for the Asian male population.

Multivariable model adjusted for age, education level, household income, physical activity, alcohol consumption, energy intake, body mass index (except for OR of the elevated WC.

Abbreviations: WC, waist circumference; TG, triglyceride; HDL-C, high density lipoprotein cholesterol; BP, blood pressure; FSG, fasting serum glucose; OR, odds ratio; NCEP, national cholesterol education program; ATP, adult treatment panel.

*p < 0.05, **p < 0.01, ***p < 0.001.

In the analysis of the association of change in smoking behavior and weight change with newly diagnosed MetS, weight gain following smoking cessation was associated with higher odds of MetS (OR = 1.90; 95% CI: 1.43–2.52). In contrast, no weight change (OR = 0.77; 95% CI: 0.60–1.00) and weight loss (OR = 0.40; 95% CI: 0.28–0.57) after smoking cessation was associated with lower odds of MetS. The main analysis of the association between change in smoking behavior, weight change, and newly diagnosed MetS is shown in Table 3. Although statistical significance was attenuated, investigation on subgroup effect, excluding those with family history, stratification by the number of components of MetS at baseline generated similar results compared to the main analysis on the association between change in smoking behavior, weight change, and newly diagnosed MetS. The results of the sensitivity analyses are summarized in Supplemental Table 1.

**Table 3:** Association of change in smoking behavior and weight change with newly diagnosed metabolic syndrome in men in the Health Examinees-Gem Study.

	Continual Smokers *(N* = *1,325)*	Smoking Cessation			Never Smokers *(N* = *1,689)*
		Quitters with weight gain^a^ (*N* = 576)	Quitters without weight change^b^ (*N* = *1,522*)	Quitters with weight loss^c^ (*N* = 697)	
**Newly Diagnosed Metabolic Syndrome** ^**d**^					
No. of events (%)	172	120	159	56	132
Age-adjusted Model	1 (referent)	1.75 (1.35–2.27)^***^	0.78 (0.61–0.98)^*^	0.58 (0.42–0.80)^**^	0.56 (0.44–0.72)^***^
Multivariable Model^e^	1 (referent)	1.90 (1.43–2.52)^***^	0.77 (0.60–1.00)^*^	0.40 (0.28–0.57)^***^	0.54 (0.42–0.71)^***^

Data presented above as adjusted OR (95% CI) using multivariable logistic regression.

^a^Quitters with weight gain of 2 kg (>+2 kg).

^b^Quitters with weight gain or loss of 2 kg.

^C^Quitters with weight loss of 2 kg (<−2 kg).

^d^Meeting three or above of the following: (1) Elevated waist circumference (WC) ≥ 90 cm; (2) High triglyceride (TG) ≥ 150 mg/dL; (3) Reduced high density lipoprotein-cholesterol (HDL-C) < 40 mg/dL or treatment with antihyperlipidemic medication; (4) High blood pressure (BP) systolic/diastolic BP ≥ 130/85 mmHg or treatment with antihypertensive medication (5) Impaired fasting serum glucose (FSG) ≥ 110 mg/dL or treatment with antihyperglycemic medication.

^e^Multivariable model adjusted for age, education level, household income, number of components of metabolic syndrome at baseline, physical activity, alcohol consumption, energy intake, body mass index, family history of hypertension, family history of type 2 diabetes, and family history of hyperlipidemia.

NOTES: Metabolic syndrome was defined by the National Cholesterol Education Program Adult Treatment Panel III modified for the Asian male population.

Weight change adjusted as a continuous variable.

*p < 0.05, **p < 0.01, ***p < 0.001.

### Secondary analysis

In secondary analyses restricting the study sample to quitters, the percentage of newly diagnosed MetS was the highest among those with weight gain (21.4%). As compared with quitters without weight change, the multivariable adjusted ORs and 95% CIs for newly diagnosed MetS among quitters with weight gain and quitters with weight loss were 2.44 (95% CI: 1.84–3.25) and 0.52 (95% CI: 0.37–0.73), respectively. The results of the secondary analysis is presented in Table 4.

**Table 4:** Association between weight change after smoking cessation and newly diagnosed metabolic syndrome among quitters.

	Smoking Cessation		
	Quitters without weight change^a^ (*N* = *1,522*)	Quitters with weight gain^b^ (*N* = 576)	Quitters with weight loss^c^ (*N* = 697)
**Newly Diagnosed Metabolic Syndrome**			
No. of events (%)	159	120	56
Age-adjusted Model	1 (referent)	2.26 (1.75–2.94)^***^	0.75 (0.54–1.03)
Multivariable Model	1 (referent)	2.44 (1.84–3.25)^***^	0.52 (0.37–0.73)^***^

Data presented above as adjusted OR (95% CI) using multivariable logistic regression model adjusted for age, education level, household income, time since cessation of smoking, number of components of metabolic syndrome at baseline, physical activity, alcohol consumption, energy intake, body mass index, uric acid, high sensitivity C-reactive protein, family history of hypertension, family history of type 2 diabetes, and family history of hyperlipidemia.

^a^Quitters with weight gain or loss of 2 kg.

^b^Quitters with weight gain of 2 kg (>+2 kg).

^C^Quitters with weight loss of 2 kg (<−2 kg).

*p < 0.05, **p < 0.01, ***p < 0.001.

## Discussion

In this community-based cohort study comprised of 5,809 middle-aged Korean men without MetS at baseline, 10.5% of the participants were newly diagnosed with MetS during the follow-up period. Weight gain following smoking cessation was significantly associated with elevated WC and impaired FSG, but no weight change and weight loss after smoking cessation were associated with improved TG and HDL-C. Among quitters, those with weight gain of more than 2 kg were more likely to develop metabolic syndrome compared to those with weight gain or loss of less than 2 kg.

One prospective study by Kim *et al*. comprised of 4,542 Korean men based on records from a single hospital found that ex-smokers had increased risk of MetS despite weight change compared to non-smokers^19^. In contrast, our study showed that only those with weight gain had higher odds of newly diagnosed MetS among quitters as compared to continual smokers. Additionally, we found that quitters with weight gain had higher odds of elevated WC and impaired FSG while quitters without weight gain had improved levels of TG and HDL-C. While both studies suggest the importance of post-cessation weight management to reduce risk of MetS, our study adds to the evidence for an association of specific components of MetS with smoking cessation by the degree of weight change.

The mechanism by which smoking cessation could reduce MetS despite weight change is not fully understood, but can be explain in part by improvement of lipid profile. Negative health effects of post-cessation weight change may be mitigated by improvements of lipid profile regardless of weight change attributable to quitting smoking. In a follow-up study of 1,955 Japanese male workers, Tamura *et al*., showed that those who had quit smoking for at least half a year had 2.0 kg of weight gain and worsening effects of BP, FSG, and total cholesterol, but they significantly improved levels of HDL-cholesterol^20^. Similarly, in another study of 226 Korean men who had quit smoking, quitters with weight gain (≥1.3 kg) had significantly harmful changes of BP, FSG, and cholesterol levels whereas quitters without weight gain (<1.3 kg) had improved levels of cardiometabolic factors^21^. These studies might have selection bias and small sample size. However, the evidence from these studies suggest that successfully abstaining from cigarette smoking may positively affect MetS as the change of post-cessation weight wanes over time^22^.

The primary strength of this study is the large sample size from the community-based cohort with verified health examination data through quality control. Moreover, the prospective cohort study design in this study limits the possibility of reverse causality as compared to the studies with cross-sectional design. Assessment of a wide range of known confounding factors associated with the study outcome also strengthens our study. In addition, evaluation of the association between smoking cessation, weight change, and MetS in quitters compared to continual smokers and a secondary analysis among quitters by the degree of weight change further strengthens our investigation. However, there are some limitations of this study that need to be acknowledged. We were not able to include women in this study due to a significantly low proportion of the middle-aged women who are smokers. Since the analytic sample was limited to the middle-aged Korean men who participated in the HEXA-G study, more studies are warranted in young adults, women, and other ethnic groups to test the generalizability of this study. Furthermore, change in smoking behavior was only assessed from the self-reported survey without biochemical assessment. In addition, variables used in our analyses that were derived from the self-reported survey (e.g. smoking status and family history of disease) should be validated in further studies.

In this community-based cohort study comprised of middle-aged men, we found that weight gain after smoking cessation was associated with higher odds of elevated WC and impaired FSG as compared with continual smoking. Quitters who gained substantial amount of weight after abstinence from smoking were more likely to develop MetS compared to the quitters without weight change. Overall, our results indicate the importance of weight control after smoking cessation to reduce the burden of MetS. Accordingly, intervention programs for weight management in clinical practice may be necessary for those who attempt to quit smoking.

## Methods

### Study population

We examined data on the participants of the Health Examinees-Gem (HEXA-G) study conducted in the Republic of Korea. The HEXA-G is a community-based multicenter study database comprised of Korean adults aged between 40 and 69 years who visited study centers for health examinations between 2004 and 2013. The HEXA-G study excluded the dataset from the sites that only participated in the pilot study, less than 2 years of follow-up, or did not pass quality control for biospecimen. The HEXA-G study has been used for research purpose previously^23–26^, and the design and validity of the study has been described in detail elsewhere^27,28^. For this study, we recruited the male participants with complete information on smoking status without MetS who were followed up to 4 years from the baseline. Female participants were not included due to the low proportion of those who reported themselves as current smokers at baseline (less than 5%). Among the 6,138 participants without MetS at baseline, we excluded those that met the following conditions: (1) missing data on smoking status (n = 36); (2) missing information on potential confounders (n = 184); (3) extremely low or high energy intake (<800 or ≥4,000 kcal/day) (n = 185). Finally, a total of 5,809 men were included in the analytic sample.

### Ethical approval and informed consent

All participants provided informed consent before enrollment to the HEXA-G study, and the dataset are publicly open only for research purpose upon request. The Institutional Review Board (IRB) of the Seoul National University Hospital, which complies with the principles of the Declaration of Helsinki, approved this study (IRB no: 1503-103-657).

### Assessment of change in smoking behavior and weight change

Based on the self-reported questionnaire and weight change from the baseline and follow-up health examination records of the HEXA-G study, we categorized the study participants as continual smokers, quitters with weight gain, quitters without weight change, quitters with weight loss, and never smokers. Post-cessation weight change category (stable, gain or loss of ≤2 kg; gain, gain of >2 kg, and loss, loss of <2 kg) was based on the previously reported average weight change attributable to smoking cessation in the middle-aged Korean men^21^.

### Assessment of metabolic syndrome

We adopted criteria from the National Cholesterol Education Program Adult Treatment Panel III (NCEP-ATP III)^29^ modified for the Asian population for the assessment of MetS in the HEXA-G study. For each participant, meeting three or more of the following conditions was defined as the MetS: (1) elevated waist circumference (WC) ≥ 90 cm; (2) high triglyceride (TG), ≥150 mg/dL or taking antihyperlipidemic drugs; (3) reduced high-density lipoprotein cholesterol (HDL-C), ≤40 mg/dL; (4) high blood pressure (BP), systolic BP ≥ 130 mmHg, diastolic BP ≥ 85 mmHg or taking antihypertensive medication; and (5) impaired fasting serum glucose (FSG), ≥100 mg/dL or taking antihyperglycemic agents.

### Assessment of potential confounders

In the HEXA-G study, information on sociodemographic factors (age, education level, and household income) was obtained from the self-reported survey. Age was categorized as 40–49 years, 50–59 years, and ≥60 years. Education level was grouped into elementary, middle/high school, college or above based on the final academic degree. Household income was divided into quartiles. We also assessed lifestyle factors (physical activity, alcohol consumption, and energy intake) from the survey data. Alcohol consumption was classified into non-drinker, abstainer (those who reported to have gradually reduce amount of alcohol consumption over time), and regular drinker (those who reported to regularly consume at least one alcoholic beverage per week). Regular exercise was defined as those who reported to routinely exercise until sweating on regular basis. Total energy intake per day (kcal/day) was estimated from converting the types and amount of servings consumed per day into macro and micro nutrient intake (grams per day) from different food categories in the food frequency questionnaire. BMI was calculated from dividing weight in kilograms (kg) by height in meters (m) squared (kg/m^2^). Data on uric acid and high sensitivity C-reactive protein were derived from the laboratory assessment, and information on family history of disease (hypertension, type2 diabetes, and hyperlipidemia) was obtained from the responses of the self-reported questionnaire.

### Statistical analysis

For the primary analysis, we chose continual smokers as the reference group as recommended by the U.S Surgeon General’s report in 1990^30^ to assess the effect of smoking cessation on MetS. We used multivariable logistic regression models to compute adjusted odds ratio (OR) and 95% confidence intervals (95% CI) to assess the association between smoking cessation, weight change, and components of MetS. We calculated age-adjusted and multivariable adjusted (adjusted for age, education level, household income, physical activity, alcohol consumption, energy intake, and BMI) ORs and 95% CIs for elevated WC, high TG, reduced HDL-C, high BP, and impaired FSG. BMI was not adjusted when computing the adjusted OR (95% CI) for elevated WC due to the high collinearity between the two variables. In addition to the variables included in the multivariable model, weight change was further adjusted as a continuous variable to examine the effect of weight change attributable to smoking cessation. For the component of MetS, participants who had already developed the metabolic alternation at the baseline were removed from the analytic sample. Similarly, we used multivariable logistic regression model adjusted for age, education level, household income, number of components of metabolic syndrome at baseline, physical activity, alcohol consumption, energy intake, body mass index, uric acid, high sensitivity C-reactive protein, family history of hypertension, family history of type 2 diabetes, and family history of hyperlipidemia to assess the relationship between smoking cessation and odds of newly diagnosed MetS. To further test whether weight change from smoking cessation alters this association, we adjusted weight change as a continuous variable in the multivariable model. For sensitivity analyses, we assessed subgroup effects by stratifying the analysis into different categories of age, education level, household income, BMI, and number of MetS components at baseline. Also, we excluded those with family history of type 2 diabetes, hypertension, and hyperlipidemia to examine whether the results remained consistent after exclusion of those with family history of disease related to MetS. In secondary analysis, we examined the association of weight change following smoking cessation only among quitters. For this analysis, we chose quitters with stable weight change (gain or loss of ≤2 kg) as the reference group, and compared the odds of newly diagnosed MetS in quitters with weight gain (gain of >2 kg) and weight loss (loss of <2 kg). All p-values were two-sided, and we considered p-value < 0.05 as statistically significant. We used SAS version 9.4 (SAS Institute Inc., Cary, NC, USA) for data collection and analyses.

## Supplementary information

Supplemental Table 1

## Data Availability

No additional data available. Only authorized researchers had access to the HEXA-G data according to the data sharing policy of Korea Centers for Disease Control and Prevention.
