# Supplementary material for: Weight change after smoking cessation and incident metabolic syndrome in middle-aged Korean men: an observational study
Source: Sci Rep. 2019 Feb 28;9:3103. doi: 10.1038/s41598-019-39811-0 (PMC6395682; doi:10.1038/s41598-019-39811-0)
Supplement: Supplementary file 1 — Supplemental Table 1 [file 41598_2019_39811_MOESM1_ESM.docx]

**[Title Page for Supplementary Information]**

**Weight change after smoking cessation and incident metabolic syndrome**

**in middle-aged Korean men: an observational study**

Running Head: *Kim et al.,* *Post-cessation* *weight change and metabolic syndrome*

Kyuwoong Kim ^1^, Seulggie Choi^1^, Jong-Koo Lee^2,3^, Ji-Yeob Choi^1^, Aesun Shin^4^,

Sue Kyung Park^1,4^, Daehee Kang^1,4,5^, Sang Min Park*^1,2^

^1^Department of Biomedical Sciences, Seoul National University Graduate School, Seoul, Republic of Korea

^2^Department of Family Medicine, College of Medicine, Seoul National University, Seoul, Republic of Korea

^3^JW Lee Center for Global Medicine, College of Medicine, Seoul National University, Republic of Korea

^4^Department of Preventive Medicine, College of Medicine, Seoul National University, Seoul, Republic of Korea

^5^Department of Environmental Medicine, Seoul National University Medical Research Center, Seoul, Republic of Korea

**Supplemental Table 1.** Sensitivity analyses for the association of change in smoking behavior and weight change with newly diagnosed metabolic syndrome.

|  |  |  | Smoking Cessation |  |  |
| --- | --- | --- | --- | --- | --- |
|  | Continual  Smokers | Quitters with  weight gain^a^ | Quitters without  weight change^b^ | Quitters with  weight loss^c^ | Never  Smokers |
| **Newly Diagnosed Metabolic Syndrome**  Subgroup Effect  Age  <50 years  ≥50 years  Education Level  Elementary  Middle/High School  College or Above  Body Mass Index  <25 kg/m^2^  ≥25 kg/m^2^  Household Income  Lower Half  Upper Half  Excluding those with family history  Without family history of type 2 diabetes  Without family history of hypertension  Without family history of hyperlipidemia  Number of components of metabolic syndrome at baseline  0  1  2 | 1 (referent)  1 (referent)  1 (referent)  1 (referent)  1 (referent)  1 (referent)  1 (referent)  1 (referent)  1 (referent)  1 (referent)  1 (referent)  1 (referent)    1 (referent)  1 (referent)  1 (referent) | 1.53 (0.89-2.63)  2.10 (1.49-2.97)^***^  2.71 (0.99-7.44)  1.63 (1.10-2.43)^*^  1.97 (1.24-3.16)^**^  1.97 (1.35-2.88)^***^  1.82 (1.20-2.77)^**^  1.83 (1.02-3.26)^*^  1.92 (1.38-2.67)^***^  1.88 (1.38-2.56)^***^  1.82 (1.31-2.55)^***^  2.01 (1.46-2.77)^***^  1.73 (0.68-4.40)  2.49 (1.58-3.93)^***^  1.48 (0.99-2.22) | 0.77 (0.48-1.24)  0.81 (0.59-1.10)  0.77 (0.28-2.09)  0.67 (0.47-0.96)^*^  0.89 (0.60-1.33)  0.64 (0.45-0.93)^*^  0.94 (0.66-1.33)  0.70 (0.42-1.16)  0.79 (0.59-1.06)  0.75 (0.57-0.99)^*^  0.82 (0.61-1.09)  0.75 (0.56-0.99)^*^  0.40 (0.15-1.07)  0.58 (0.37-0.91)^*^  0.93 (0.67-1.30) | 0.20 (0.08-0.45)^***^  0.47 (0.32-0.70)^***^  0.40 (0.09-1.66)  0.41 (0.26-0.66)^***^  0.35 (0.20-0.61)^***^  0.20 (0.10-0.41)^***^  0.61 (0.40-0.92)^*^  0.37 (0.18-0.76)^**^  0.40 (0.27-0.60)^***^  0.37 (0.25-0.54)^***^  0.42 (0.28-0.63)^***^  0.44 (0.30-0.65)^***^  0.34 (0.09-1.32)  0.32 (0.17-0.60)^***^  0.49 (0.29-0.69)^***^ | 0.41 (0.25-0.67)^***^  0.69 (0.43-0.81)^**^  0.38 (0.13-1.11)  0.60 (0.41-0.87)^**^  0.45 (0.30-0.69)^***^  0.48 (0.33-0.71)^***^  0.64 (0.45-0.92)^*^  0.53 (0.30-0.93)^*^  0.54 (0.40-0.73)^***^  0.49 (0.37-0.66)^***^  0.55 (0.40-0.75)^***^  0.55 (0.40-0.75)^***^  0.49 (0.20-1.17)  0.43 (0.27-0.69)^***^  0.59 (0.41-0.83)^**^ |

Data presented above as adjusted OR (95% CI) using multivariable logistic regression model presented in Table 3.

NOTES: Metabolic syndrome was defined by the National Cholesterol Education Program Adult Treatment Panel III modified for the Asian male population.

^a^Quitters with weight gain of 2kg (>+2 kg)

^b^Quitters with weight gain or loss of 2kg

^C^Quitters with weight loss of 2kg (<-2 kg)

*p<0.05, **p<0.01, ***p<0.001
